# Dupilumab improves clinical symptoms in children with Netherton syndrome by suppressing Th2-mediated inflammation

**DOI:** 10.3389/fimmu.2022.1054422

**Published:** 2022-12-08

**Authors:** Shi Yan, Xuege Wu, Jinqiu Jiang, Shijuan Yu, Xiao Fang, Huan Yang, Xiaoming Bai, Hua Wang, Xiaoyan Luo

**Affiliations:** ^1^ Department of Dermatology, Children’s Hospital of Chongqing Medical University Clinical Research Center for Child Health and Disorders, Ministry of Education Key Laboratory of Child Development and Disorders, Chongqing, China; ^2^ Chongqing Key Laboratory of Child Infection and Immunity, Children’s Hospital of Chongqing Medical University, Chongqing, China; ^3^ Ministry of Education Key Laboratory of Child Development and Disorders, Children’s Hospital of Chongqing Medical University, Chongqing, China

**Keywords:** Netherton syndrome, *SPINK5* gene, LEKTI, dupilumab, treatment

## Abstract

**Background:**

Netherton syndrome is a rare, life-threatening autosomal recessive genetic disorder with no effective treatment yet. Skin barrier dysfunction caused by *SPINK5* gene mutations is a hallmark of the disease. Antigen penetration through the defective skin and nonspecific inflammation provide a pro-T helper 2 (Th2) immune microenvironment in the disease. Therefore, Th2 cytokines are considered to be candidate therapeutic targets.

**Objective:**

To evaluate the clinical responses of patients with Netherton syndrome to dupilumab, an IL-4Rα antagonist, and identify changes in the Th1/2/17 pathway activity, skin barrier defect protein LEKTI expression after treatment.

**Methods:**

Four children with severe Netherton syndrome (aged 2 y to 4 y and 6 m) who were treated with dupilumab from January to June 2022 were evaluated at baseline, and at 4, 8, 12, 16, and 20 weeks after the start of dupilumab administration. Treatment response was assessed using the Eczema Area and Severity Index (EASI), the Numerical Rating Scale (NRS), the Dermatology Life Quality Index (CDLQI), and the Dermatitis Family Impact-questionnaire (DFI). Blood eosinophil counts, serum IgE levels and inflammatory cytokines were measured. The immunotyping of Th1/2/17 cells was performed by flow cytometry and cytokine expressions in T cell subsets were analyzed by single-cell RNA sequencing. In addition, expression of the LEKTI in skin lesions was evaluated by immunohistochemical analysis.

**Results:**

All four patients experienced clinical improvement, with significantly reduced EASI scores (by 75.0–83.9%) and NRS (by 87.5–90.0%) from baseline to 20 weeks of treatment. Improved quality of life scores were also seen for all patients, as measured by CDLQI and DFI. Serum IgE levels also fell by 75.6–86.9%. The serum Th2 cytokines IL-4, IL-5 and IL-13 were found at low level, with no significant changes during the treatment. However, Th2 cytokines expressed by T cells, especially IL-4, decreased at single-cell level after treatment (*P* = 0.029). The baseline percentage of Th2 cells (among total CD3^+^CD4^+^ T cells) was significantly higher in patients than that in healthy controls (HC) (*P* < 0.0001); this percentage fell from 8.25% ± 0.75% to 4.02% ± 0.62% after 20 weeks dupilumab treatment. There was no noticeable change in LEKTI protein expression in skin lesions pre- and post-treatment. Two patients reported mild ocular adverse effects, but there were no severe adverse events.

**Conclusion:**

Dupilumab may be an effective and safe treatment option in a subset of pediatric patients with Netherton syndrome, especially in improving itch and the quality of life. These effects were achieved in part by suppression of the Th2-mediated inflammation.

## Introduction

Netherton syndrome (NS; OMIM#256500), also known as Comel-Netherton syndrome, is a rare autosomal recessive disorder caused by germline loss-of-function mutations in *SPINK5* gene. The disorder is characterized by the classical triad symptoms of congenital ichthyosis, bamboo hair, and atopic diathesis with high serum IgE level (1). In most *SPINK5* mutations, premature stop codons result in a truncated Lymphoepithelial Kazal-type-related inhibitor (LEKTI) protein which leads to an unrestricted activity of kallikrein (KLK)-related peptidases (KLK5, KLK7, and KLK14). This will cause severe skin barrier breakdown and secondary inflammation (2, 3).

NS is a multisystemic disease for which a safe and effective treatment is not yet available. Several novel systematic treatments have been suggested in the recent literature, including immunoglobulins and biologicals that target specific inflammatory pathways (4). Dupilumab, a monoclonal antibody that blocks the IL-4 receptor (IL-4R), has been used to treat moderate to severe atopic dermatitis with a good safety and efficacy profile (5). A few recent case report displayed clinical effect with improving skin symptoms in patients with NS who treated with dupilumab (6–12). However, these reports have shown great heterogeneity in outcome measures and a few cases reported long-term outcomes. In addition, few reports describe the efficacy and safety of dupilumab in pediatric NS patients. In this prospective case series study, we reported four pediatric NS patients treated with dupilumab and their clinical responses, cytokine profiles change with treatment, which hopes to provide new insight into the underlying mechanisms of dupilumab therapy in NS and provide more evidence to support biologic treatment for NS patients.

## Methods

### Patients and control subjects

Four NS patients (median age, 3.25y; range from 2y to 4.5y) from four unrelated Chinese families were enrolled. A diagnosis of NS was made based on clinical signs, *SPINK5* mutations, and decreased/absent LEKTI expression upon immunostaining analysis. Health controls (HCs) comprised eight age-matched subjects (median age, 3 y; range from 1.5y to 5y). Patients were evaluated, and blood was collected at baseline (before therapy), and at 4, 8, 12, 16 and 20 weeks after dupilumab administration. Skin biopsies were collected at baseline and 20 weeks. All parents provided written informed consent, and the study was approved by the Ethics Committee of the Children’s Hospital of Chongqing Medical University (2021-17).

### Treatment

All NS patients received a loading dose of subcutaneously administered dupilumab (400 mg), followed by 200 mg every 4 weeks for 20 weeks. During dupilumab treatment, patients were encouraged to continue the use of moisturizers, topical corticosteroids, or 1% Pimecrolimus, which were not monitored specifically.

### Measures used in this study

The Children’s Dermatology Life Quality Index (CDLQI) and the Dermatitis Family Impact-questionnaire (DFI) were adopted for evaluation of quality of life, the Eczema Area and Severity Index (EASI), and the Numerical Rating Scale (NRS) were used at every visit for assessing changes in symptoms. Laboratory tests, including total serum immunoglobulin (Ig) E and eosinophil (ESO) counts, were conducted at every visit. A high level of serum IgE was defined as >165.0 IU/ml, and eosinophilia was defined as a blood eosinophil count >0.68×10^9^ cell/L.

### Assessment of serum cytokine profiles

Multiple cytokines, including IL-1 *β*, IL-1*α*, TNF- *α*, TNF- *β*, IFN-*γ*, IL-2, IL-4, IL-5, IL-6, IL-8, IL-9, IL-12p70, IL-13, IL-17A, IL-23, and IL-31, were measured at baseline, 4 and 20 weeks after treatment with dupilumab by Luminex 200™ System (Millipore) based on manufacturer’s instructions. Serum samples were diluted 2 times and incubated for 3 hours with a mix of beads coupled to antibodies specific for each measured cytokine. Then a mixed secondary streptavidin-coupled antibodies was added for 1 hour followed by phycoerythrin (PE)-conjugated biotin after several washing steps. The signal intensity was measured by PE fluorescence and a standard curve was performed to identify the absolute concentration of each cytokine in tested samples.

### Assessment of cytokine profiles in T cell subsets by single-cell RNA sequencing

The PBMCs from 4 NS patients at baseline and 20 weeks after dupilumab treatment were sequenced for single cells using the 10x Genomics scRNA-seq platform. Based on the single-cell transcriptomic data, Cytokine Signaling Analyzer (CytoSig; https://cytosig.ccr.cancer.gov/) was employed in T cell subsets to evaluate the cytokine signaling activity, including IL-1A, IL-1B, IL-2, IL-4, IL-10, IL-12, IL-13, IL-15, IL-17A, IL-21, and IL-22.

### Flow cytometry analysis and antibodies

Peripheral blood mononuclear cells (PBMCs) were isolated from blood samples using Ficoll-Hypaque (GE Healthcare, USA) gradient centrifugation. To obtain Th1/2/17 cells, PBMCs were incubated for 30 min in with specific antibodies (FITC-anti-CD45RA, PE-anti-CCR6, Percep-anti-CD3, PE/Cy7-anti-CD4, APC-anti-CXCR3, BV421-anti-CXCR5, BV510-anti-CCR4; all from BD Biosciences) in PBS containing 2% fetal bovine serum. Cells were examined immediately using a BD FACS-CantoII, and data were analyzed using FlowJo software (TreeStar, USA). Cells were classified as follows: Th17 cells, CD3^+^CD4^+^CD45RA^-^CXCR5^-^CCR6^+^CCR4^+^; Th2 cells, CD3^+^CD4^+^CD45RA^-^CXCR5^-^CCR6^-^CCR4^+^CXCR3^-^; and TH1 cells, CD3^+^CD4^+^CD45RA^-^CXCR5^-^CCR6^-^CCR4^-^CXCR3^+^.

### Immunohistochemistry

After consent was obtained from the parents, skin biopsy was conducted in the ichthyosis lineariz circumflexa (ILC) lesions of patients 2 and 3. Four normal skin specimens obtained from routine surgery were served as controls. Skin sections were fixed, sectioned transversely (4*μm*), and embedded in paraffin blocks. The slides were permeabilized with 0.5% H_2_O_2_, then stained with a SPINK5 polyclonal antibody (rabbit IgG; Invitrogen, PA5-52820) overnight at 4°C. A secondary biotinylated antibody (ZSGB-BIO, PV-9001, China) was performed, then reacted with diaminobenzidine (DAB) (ZSGB-BIO, ZLI-9018, China). After counterstaining with hematoxylin, images were captured under a digitalized brightfield microscope (Leica Imaging Systems Ltd., Cambridge, U.K.) and analyzed with ImageJ software (version 2.1.0). Average optical density (AOD) was used to evaluate immunofluorescence staining.

### Statistical analysis

Statistical analyses were performed using GraphPad Prism software, version 8.3.1 (GraphPad Software, San Diego, CA, USA). A two-tailed unpaired t-test was used for single comparisons, and the Wilcoxon matched pairs signed rank test or the Mann-Whitney test was used to assess the statistical significance of differences between groups (****P < 0.0001; ***P < 0.001; **P < 0.01; and *P < 0.05).

## Results

### Demographics and genetics

The characteristics of the four NS patients (two males and two females; average age, 3.25 ± 1.041y; range, 2y–4.5y) are shown in the **Table 1**. All of the patients presented with ILC, Trichorrhexis Invaginata or bamboo hair (**Supplement Figure S1**), growth retardation, and high levels of serum IgE. Whole exome sequencing of peripheral blood DNA identified *SPINK5* mutations in all patients. (**Supplement Figure S2**) Two novel heterozygous mutations were found in patient 3 (IVS10+5G>T; c.2258_59insA, exon24), and patient 4 (c.2143dupA, exon23).

**Table 1:** Clinical and genetic characteristics of the patients with Netherton syndrome.

Patient	Age	Gender	Weight(kg)	Height(cm)	ILC	TI	Food allergy	Growth retard	IgE(IU/ml)	*SPINK5* mutation	Mutation-type
1	3y6m	F	13	86	**+**	**+**	–	**+**	713↑	exon26, c.2474_75delAG (p.E825Gfs*2)	Hom
2	3y	F	11	80	**+**	**+**	–	**+**	3330↑	exon27, c.2557C>T (p.R853X) exon1-34, lossl (73K)^#^	Het Het
3	2y	M	7	70	**+**	**+**	**+**	**+**	5870↑	IVS10+5G>T^#^ exon24, c.2258_59insA (p.R753Rfs*4)^#^	Het Het
4	4y6m	M	12.5	94	**+**	**+**	–	**+**	6940↑	exon23, c.2143dupA (p.N716Kfs*11)^#^ exon25, c.2423C>T (p.T808I)	Het Het

M, month(s); Y, year(s); F, female; M, male; ILC, Ichthyosis Linearis Circumflexa; TI, Trichorrhexis Invaginata; Hom, homozygous; Het, heterozygous; SPINK5:Serine Peptidase Inhibitor Kazal Type5; + positive; - negative; ^#^indicates unreported SPINK5 mutation, the red ↑ represents the abnormal (elevated) level.

### Dupilumab significantly improved skin lesions, itch, and quality of life

**Figure 1:**
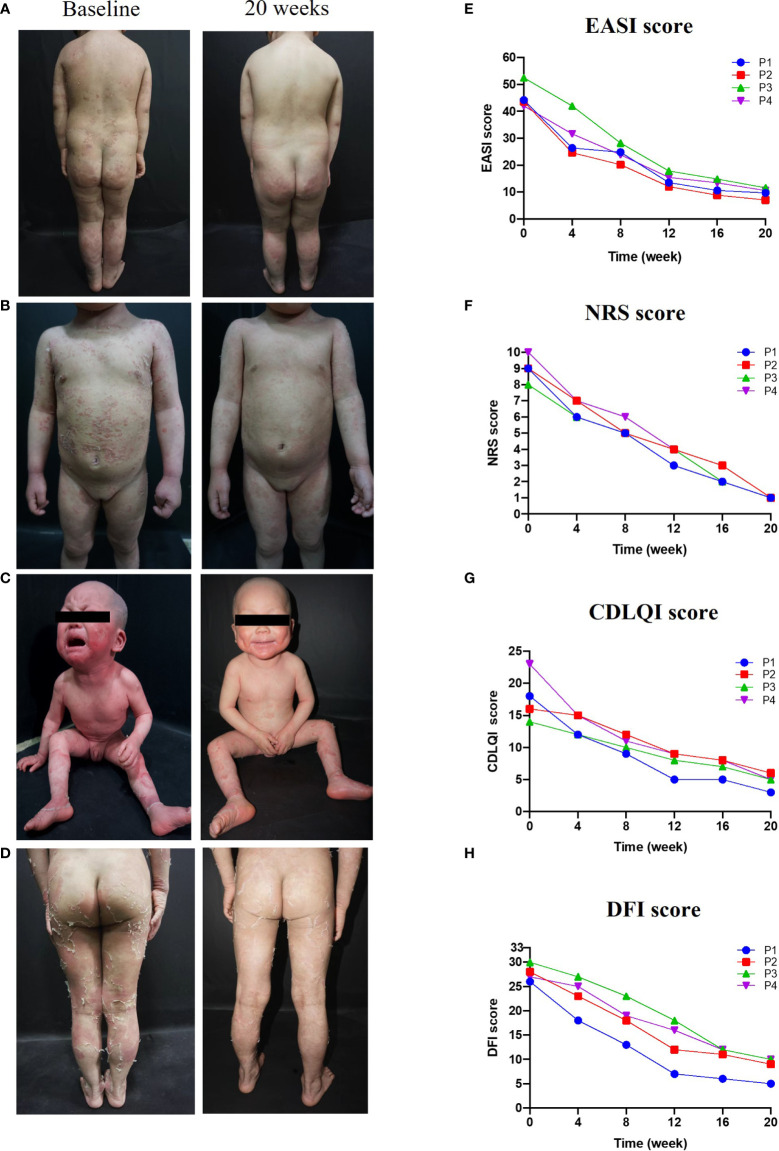
Improvements in clinical symptoms and QoL after 20 weeks of dupilumab treatment Images in **(A–C)**, and **(D)** show clinical pictures of patients 1,2,3, and 4 before and 20 weeks after treatment with dupilumab, respectively. **(E–H)** displayed changes in disease severity, pruritus, and QoL over time of treatment, **(E)** Eczema Area and Severity Index (EASI), **(F)** the Numerical Rating Scale (NRS), **(G)** the Dermatology Life Quality Index (CDLQI), and **(H)** the Dermatitis Family Impact-questionnaire (DFI).

Treatment with dupilumab led to a significant improvement in clinical signs (see the images in **Figures 1A–D**). The affected area of skin lesions decreased to varying degrees: 75% in patients 1 and 3, and 50% in patients 2 and 4. The disease severity and pruritus were reduced markedly at 20 weeks post-dupilumab treatment, evidenced primarily by changes in the EASI and NRS (**Figures 1E, F**). The four patients reported at least 75.0% improvement in the EASI from baseline and an 87.5% reduction in the NRS. Accordingly, the life quality improved as CDLQI and DFI decreased by 72.1% (mean value) and 69.6% (mean value) from baseline to Week 20, respectively (**Figures 1G, H**). We also observed continued weight gain along the third percentile in all four patients compared to baseline. Interestingly, the height of patient 3 increased by 5 cm during treatment, after failure to thrive 1 year before treatment. Hair growth seemed to occur only in patient 1 and hair shaft nodules were not changed in any of our patients.

### Dupilumab significantly decreased serum IgE in NS

**Figure 2:**
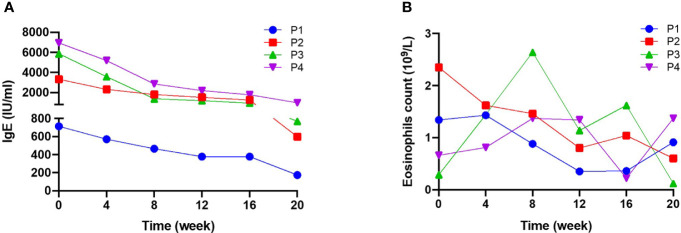
Serum IgE levels and blood eosinophil counts during 20 weeks of dupilumab treatment **(A)** serum IgE levels and **(B)** blood eosinophil (EOS) count were measured for 20 weeks. Serum IgE levels fell significantly after treatment, while there were no significant changes in EOS.

All four patients had high serum IgE, and two had eosinophilia (patients 3 and 4) at baseline. During treatment, serum IgE levels fell steadily in all patients (**Figure 2A**): from 713 to 174 IU/ml (a decrease of 75.6%) in patient 1, from 3330 to 598 IU/ml (a decrease of 82.0%) in patient 2, from 5870 to 769 IU/ml (a decrease of 86.9%) in patient 3, and from 6940 to 988 IU/ml (a decrease of 85.8%) in patient 4. In addition, the EOS count in three patients fell slightly from the baseline value (**Figure 2B**). However, it is noteworthy that a transient elevation in the EOS count was observed over the course of dupilumab treatment, although the count was lower at Week 20 than at baseline.

### Dupilumab suppressed Th2-type immune responses in NS

**Figure 3:**
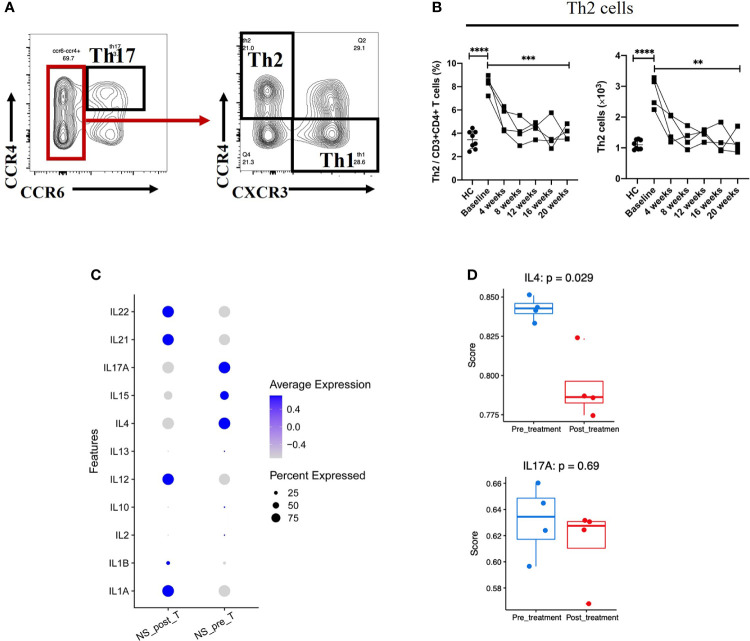
Reduction in Th2-mediated inflammation after 20 weeks of dupilumab treatment **(A)** Gating strategy used to identify Th2 and Th17 cells. **(B)** Changes in the percentages of Th2 cells among the CD3+CD4+ T cell population and in the absolute number of Th2 cells from baseline to 20 weeks of dupilumab treatment. **(C)** Dot plot illustrating (the top 11/select key) cytokine expression signatures/patterns of T cells before (pre) and 20 weeks after (post) dupilumab treatmentthe color intensity indicates the relative expression level and the size of the dots represents the percentage of cells expressing the cytokine genes across T cell subsets. **(D)** Quantitative scatter plot with a bar graph revealed the expression level of IL-4 and IL-17A in T cells across treatment conditions. The Wilcoxon matched pairs signed rank test or the Mann-Whitney U test was used to assess statistical significance: ****P < 0.0001; ***P < 0.001; **P < 0.01. Each dot represents a value for each patient.

Although serum Th2 cytokines were at the normal range or low amounts (**Table 2**), the percentage (among total CD3+CD4+ cells) and absolute number of Th2 cells in NS patients was much higher than that in HCs (*P* < 0.0001) (**Figures 3A, B**). Moreover, the percentage of Th2 cells (among total CD3+CD4+ cells) fell from 8.25%± 0.75% to 4.02%±0.62% (mean ± SD), and the absolute number of Th2 cells was decreased significantly after 20 weeks of treatment (*P* = 0.0021). The numbers of Th17 and Th1 cells, however, tended to increase slightly, but the changes had no statistically significance (**Supplement Figures S3A, B**). Next, we examined the Th2/Th17 balance and found that the Th2/Th17 ratio was significantly lower after dupilumab therapy (*P* = 0.0128) (**Supplement Figure S3C**), suggesting that dupilumab might counterbalance the skew toward Th2 responses. In addition, dupilumab treatment down-regulated the expression of IL-4, a pivotal type 2 cytokines, at single cell level (*P* = 0.029). (**Figures 3C, D**)

**Table 2:** Serum cytokine changes after 20 weeks of dupilumab treatment.

		IL-1*β*	IL-*1α*	TNF- *α*	TNF- *β*	IFN-*γ*	IL-2	IL-4	IL-5	IL-6	IL-8	IL-9	IL-12p70	IL-13	IL-17A	IL-23	IL-31
				(pg/ml)	(pg/ml)	(pg/ml)	(pg/ml)	(pg/ml)	(pg/ml)	(pg/ml)	(pg/ml)	(pg/ml)	(pg/ml)	(pg/ml)	(pg/ml)	(pg/ml)	(pg/ml)	(pg/ml)	(pg/ml)
**P1**	Baseline	< 1.2	< 0.4	13.5	< 1.9	9.3	< 3.6	< 5.5	< 3.6	207.6↑	190.5↑	< 2.7	< 3.6	2.6	< 0.8	< 4.4	< 2.3
	4w	< 1.5	< 0.6	< 3.4	< 4.9	< 4.7	< 3.9	< 5.4	< 5	< 7.6	12.5	< 3.2	< 5.8	< 2	< 1.1	< 6.6	< 4.1
	20w	1.3	< 0.5	< 3.1	< 3	< 5.2	< 4.1	< 7.4	< 5.4	< 7.7	19.4	< 4.7	< 4.3	< 3.4	< 1.5	< 4.2	< 4.9
**P2**	Baseline	11.7	< 0.4	< 2.5	< 1.7	13	< 4.4	5.9	< 5.3	< 5.9	286.8↑	< 2.5	< 4.2	1.8	1	< 3.1	< 1.9
	4w	4.5	< 0.6	< 3.4	< 4.9	80	4.6	< 5.4	< 5	< 7.6	165.6↑	< 3.2	< 5.8	2.5	< 1.1	< 6.6	< 4.1
	20w	< 0.9	< 0.5	< 3.1	< 3	< 5.2	< 4.1	< 7.4	< 5.4	< 7.7	81.5↑	< 4.7	< 4.3	< 3.4	< 1.5	< 4.2	< 4.9
**P3**	Baseline	< 1.3	< 0.4	< 2.9	< 2.5	< 4.7	< 3.2	< 5.8	< 4.3	201.2↑	74.9↑	< 2.3	< 6	< 1	< 1.3	< 6.5	< 1.5
	4w	1.6	< 0.6	< 3.4	< 4.9	34.5	< 3.9	< 5.4	< 5	8.6	29.5	< 3.2	< 5.8	< 2	< 1.1	< 6.6	< 4.1
	20w	6.8	< 0.5	22.1	< 3	< 5.2	< 4.1	< 7.4	< 5.4	< 7.6	< 2	< 4.7	< 4.3	< 3.4	< 1.5	< 4.2	< 4.9
**P4**	Baseline	1.7	1.1	6.2	< 1.6	< 8.8	< 3.9	< 6	< 5.7	222.7↑	124.2↑	< 2.2	< 3.6	< 1.9	< 2.4	< 3.3	< 5.5
	4w	< 1.5	2.8	< 3.4	< 4.9	7.1	< 3.9	< 5.4	< 5	124.2↑	64.4↑	< 3.2	< 5.8	< 2	< 1.1	< 6.6	< 4.1
	20w	4.4	< 0.5	3.1	< 3	< 5.2	< 4.1	< 7.4	< 5.4	< 7.6	25.7	< 4.7	< 4.3	< 3.4	< 1.5	< 4.2	< 4.9

The values and ↑ in red represents the abnormal (elevated) level.

### No obvious changes of LEKTI expression in skin lesions

**Figure 4:**
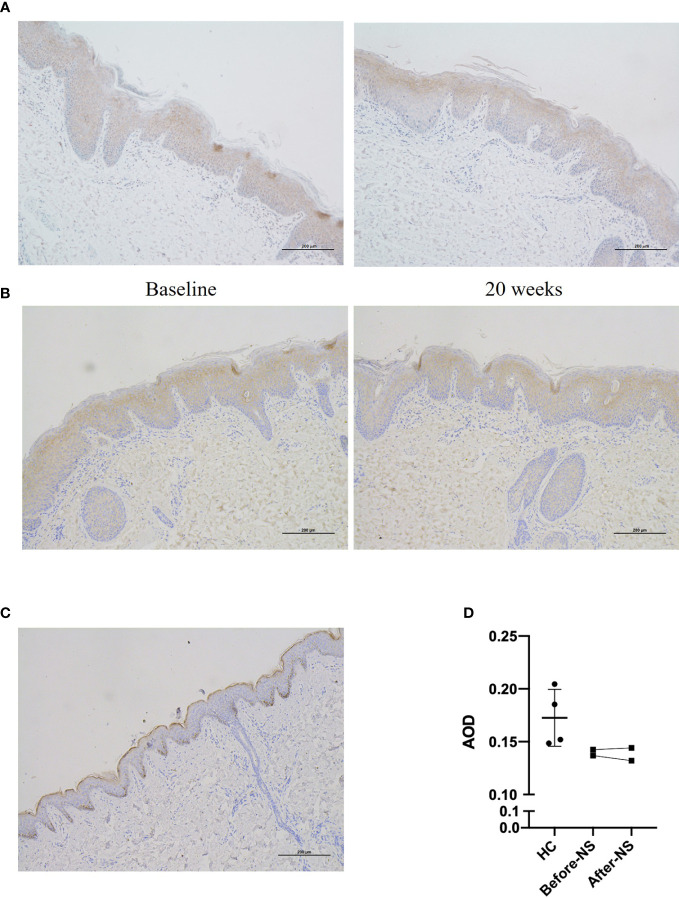
Expression of LEKTI protein in skin lesions of patients after 20 weeks of dupilumab treatment **(A, B)** show immunohistochemical staining of skin lesion for LEKTI before and after 20 weeks of dupilumab treatment in patients 1 and 4, respectively. Both patients showed a significant reduction in LEKTI expression compared with that of HCs. **(C)** LEKTI expression in HCs as a positive control: LEKTI is expressed strongly in the cytoplasm of keratinocytes in the epidermal granular and uppermost spinous layers. **(D)** Average optical density (AOD) was analyzed. The results suggest that LEKTI expression was significantly lower in the lesion skin of both patients than that of HCs and there was no obvious change after dupilumab treatment.

Skin samples from patient 1 and patient 4 showed diminished expression of LEKTI which displayed in HC, however, as a continuous staining of the cytoplasm in their epidermal granular layer and uppermost spinous layers (**Figure 4**). Furthermore, LEKTI expression in skin lesions of patient 1 and patient 4 did not change after 20 weeks of treatment, as expected.

### Safety

During the entire 20-week treatment period, two patients reported mild ocular symptoms presented with bilateral conjunctival hyperemia, pruritus, tearing and foreign-body sensation and alleviated by using tobramycin and dexamethasone eye drop, which did not lead to treatment discontinuation. No other commonly reported treatment-related side effects were observed.

## Discussion

NS is a rare genetic disease and the existing treatment options are mostly symptomatic. Reports on the clinical effects of biologicals on NS are scarce. In the current prospective case series study, four pediatric NS patients treated with dupilumab have achieved an impressive and sustained responses, with a marked improvement in skin-related symptoms, pruritus, and quality of life. In addition, the frequency of their topical corticosteroid use was reduced, thus reducing the risk of severe local and systemic side effects, such as Cushing syndrome and growth retardation. We employed standardized measurement instruments to evaluate the treatment outcomes, therefore, avoided evidence of poor quality and heterogeneity in reporting outcomes in previous studies.

The core pathogenesis of NS is the loss of protease inhibitor LEKTI, which causes severe skin barrier impairment and triggers the expression of proinflammatory and proallergic cytokines by activating protease-activated receptor-2 (PAR-2) signaling in keratinocyte (13). Upregulated inflammatory pathways, including Th2 and Th17, and elevated TNF-α, TSLP and IgE levels in patients with NS have been observed and several biologicals targeting IL-4, IL-12/23, IgE, TNFα and IL-17 have been tried (4). The results demonstrated that both biologicals targeting Th2 and Th17 pathways appear to be effective in NS, however, the potential mechanisms are not clear (4). A recent research using transcriptomic and proteomic analysis in 13 adult NS patients revealed that IL-17/IL-36 pathways were predominantly activated axes and Th17-driven immune response was mainly involved the NS pathogenesis (14). However, the clinical benefits with using anti-IL-17A antibody for part of NS patients were reported not sustained for long (15), and most NS patients were accompanied by severe atopic diathesis with very high serum IgE levels and allergic responses to environment triggers. In addition, the molecular profiling in above mentioned multiomic study revealed a Th2 predominant signature in a subset of patients characterized by ichthyosis linearis circumflflexa (ILC) (14), suggesting that other biological pathways, including Th2 axis, may be involved in NS pathogenesis.

In present study, 4 pediatric NS patients presented with ILC phenotype, one of them displayed erythroderma at his early age. Immunophenotyping of PBMCs at the baseline revealed increased Th2 subset in all patients, but only slightly increased Th17 cell numbers. After dupilumab treatment, except for sustained symptomatic improvements, a significant decrease in both percentage (among total CD3+CD4+ cells) and absolute number of Th2 cells were observed, suggesting that the Th2 pathway activation contributed to the systemic inflammation in our cohort. This hypothesis is supported by increased IL-4 production in T cell subsets, which could in turn induces B cell proliferation, isotype switching and IgE production (16). What is evident is that after blocking the IL-4Rα by using dupilumab, the elevated serum IgE levels fell steadily in all patients. Although serum IL-4 and IL-5 were detected at normal range as the previous study (17), the serum IL-6 and IL-8 were found to be significantly elevated and followed by a gradual decline during the treatment with dupilumab. Studies have shown that IL-6 plays a key role in adaptive immune response, which promotes the maturation of B cells and is considered to be one of the necessary cytokines to induce B cells to secrete IgE antibodies (18, 19), but how it is involved in mediating IgE production in NS patients is unclear. Of note, as a member of the CXC family, IL-8 is primarily responsible for chemotaxis neutrophils into inflammatory tissues (20). This is in good concordance with the recent finding that marked neutrophil skin infiltration in ILC type NS (14). However, the specific role and underlying mechanisms of IL-8 in neutrophil skin infiltration in NS remain to be studied.

We further evaluated the LEKTI protein expression in skin lesions of the NS patients in pre-and-post dupilumab therapy. Unsurprisingly, there were no obvious changes in LEKTI expression were observed, indicating that the improvements in skin lesions by dupilumab mainly rely on suppression of downstream Th2-mediated inflammation, rather than to regulate or correct underlying LEKTI defects. In regarding to the drug safety, only two patients in this study have reported mild ocular symptoms, which similar to that in treatment of atopic dermatitis, in the entire 20-week treatment period. It goes without saying that dupilumab has accumulated a lot of experience and safety profiles in pediatric population, especially it has recently been approved for children up to 6 months of age (21). Contrarily, IL-17A biologicals Secukinumab and Ixekizumab have not approved for children under 6 years and the safety data for children and infants are scarce. When we face an infant with NS, from the perspective of drug safety, dupilumab has obvious superiority.

Taken together, our results suggest that dupilumab is a safe and effective treatment option for certain subtype of pediatric NS patients, especially with respect to in improving skin symptoms, itching and the quality of life. The possible mechanisms may involve the suppression of the Th2-mediated inflammation, but further studies with larger samples are needed.

## Limitations

The study has a small number of recruited patients and the molecular features in skin lesion were not detected, which should be taken into account when drawing conclusions.

## Data availability statement

The datasets presented in this study can be found in online repositories. The names of the repository/repositories and accession number(s) can be found below: GEO submission, accession number(GSE214985).

## Ethics statement

The studies involving human participants were reviewed and approved by the Ethics Committee of the Children’s Hospital of Chongqing Medical University. Written informed consent to participate in this study was provided by the participants’ legal guardian/next of kin. Written informed consent was obtained from the individual(s), and minor(s)’ legal guardian/next of kin, for the publication of any potentially identifiable images or data included in this article.

## Author contributions

Concept and design: XL, HW, SYu, and XB. SYa and XL had full access to all of the data in the study and take responsibility for the integrity of the data and the accuracy of the data analysis. Drafting of the manuscript: SYa, XL, HW and XB. All authors participate in patient enrollment and follow-up.

## Supplementary material

Supplementary Figure 1 Observations of hair from the patient 1 under dermoscopy and light microscopy Hair shaft shows typical bamboo nodes under the dermoscopy **(A)** and light microscopy **(B)**.

Supplementary Figure 2 Genetic characterization of the patients. **(A)** Family pedigrees of the patients. Male subjects, squares; female subjects, circles; patient, black squares/circles; proband, arrows. **(B)** Sequence analysis of the *SPINK5* gene. The mutations are indicated by the arrow.

Supplementary Figure 3 Changes in Th1/17 cell subsets during 20 weeks of dupilumab treatment Changes in the percentage of Th17 cells **(A)** and Th1 cells **(B)** among the CD3+CD4+ T cell population and in the absolute number of Th17 cells **(A)** and Th1 cells **(B)** from baseline to 20 weeks of treatment. **(C)** Changes in the Th2/Th17 ratio from baseline to 20 weeks of treatment. The Wilcoxon matched pairs signed rank test or the Mann-Whitney U test was used to assess statistical significance: ****P < 0.0001; ***P < 0.001; **P < 0.01; and *P < 0.05. Each dot represents a value for each patient.
