# Supplementary figures and images for: Dupilumab improves clinical symptoms in children with Netherton syndrome by suppressing Th2-mediated inflammation
Source: Front Immunol. 2022 Dec 8;13:1054422. doi: 10.3389/fimmu.2022.1054422 (PMC9773867; doi:10.3389/fimmu.2022.1054422)

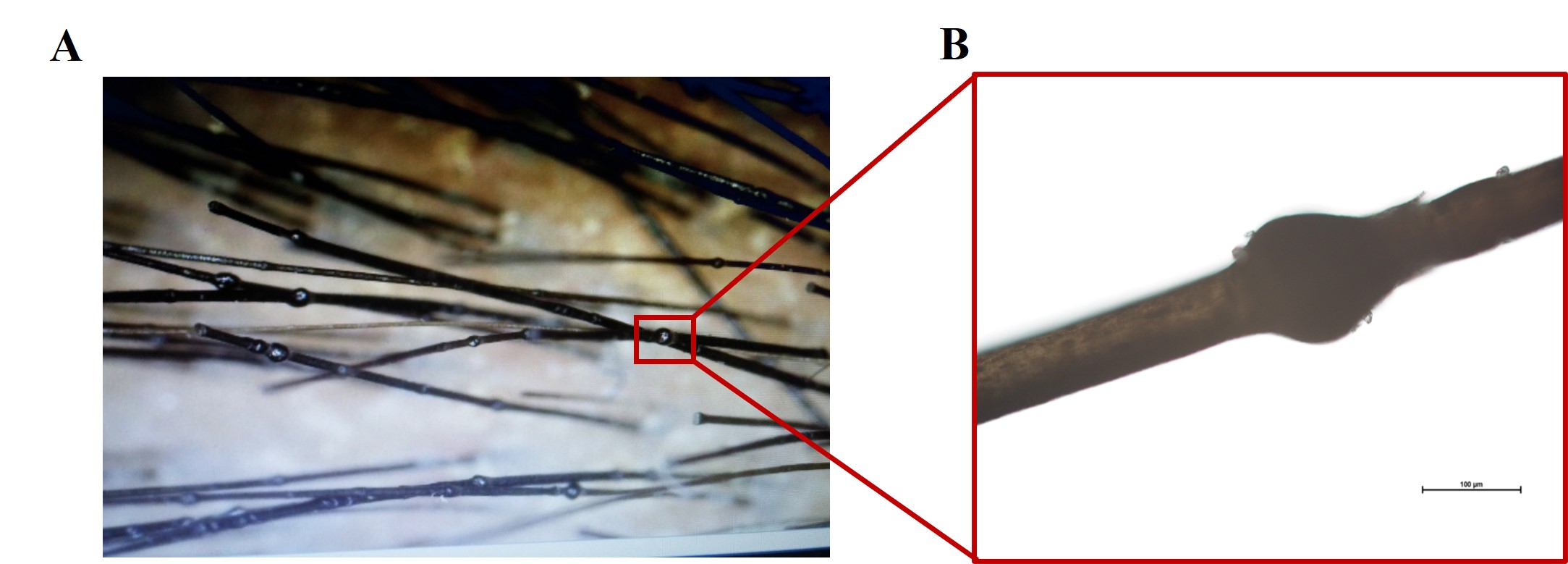

Supplement: Supplementary Figure 1 — Observations of hair from the patient 1 under dermoscopy and light microscopy Hair shaft shows typical bamboo nodes under the dermoscopy (A) and light microscopy (B). [file Image_1.jpeg]

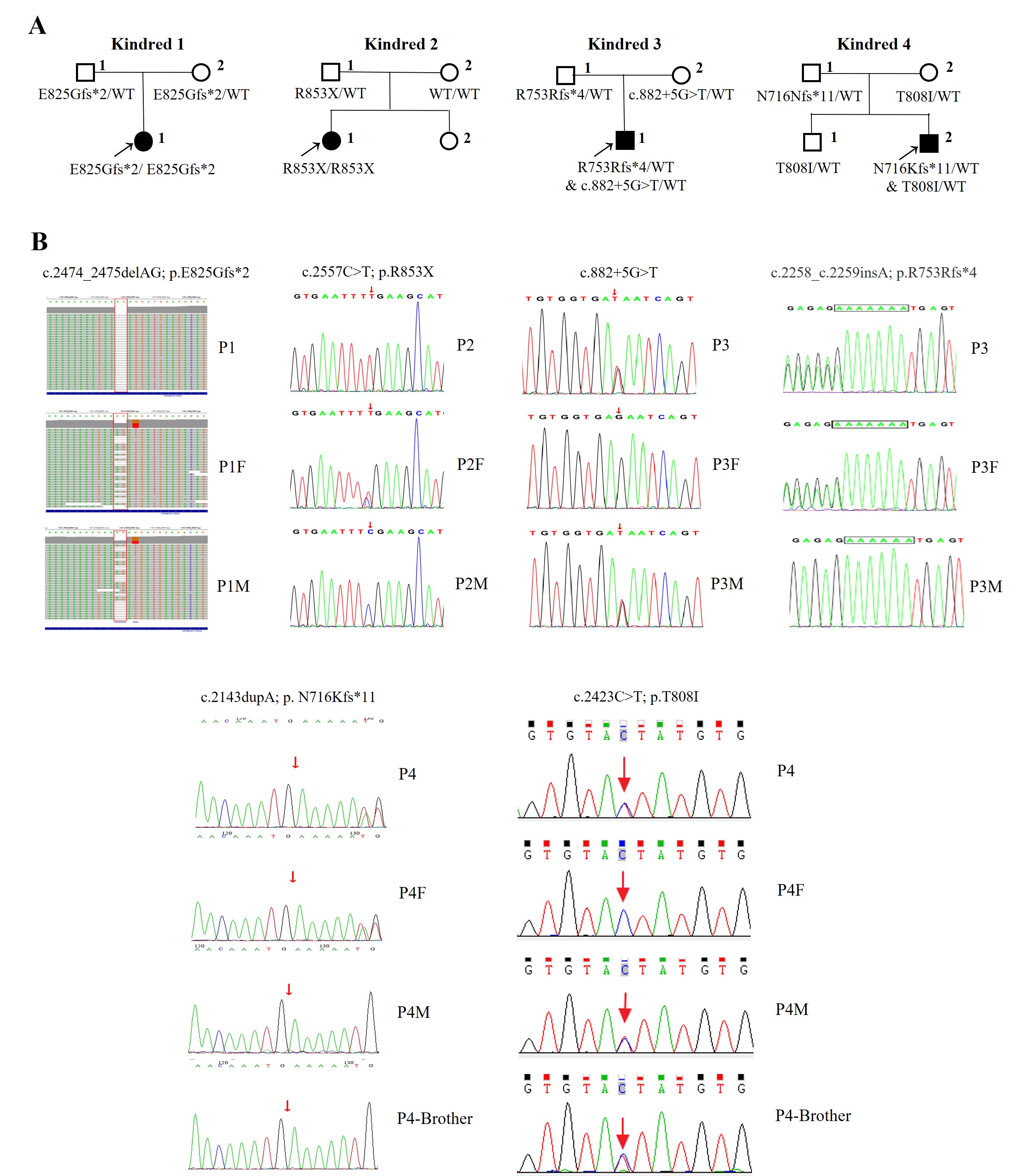

Supplement: Supplementary Figure 2 — Genetic characterization of the patients. (A) Family pedigrees of the patients. Male subjects, squares; female subjects, circles; patient, black squares/circles; proband, arrows. (B) Sequence analysis of the SPINK5 gene. The mutations are indicated by the arrow. [file Image_2.jpg]

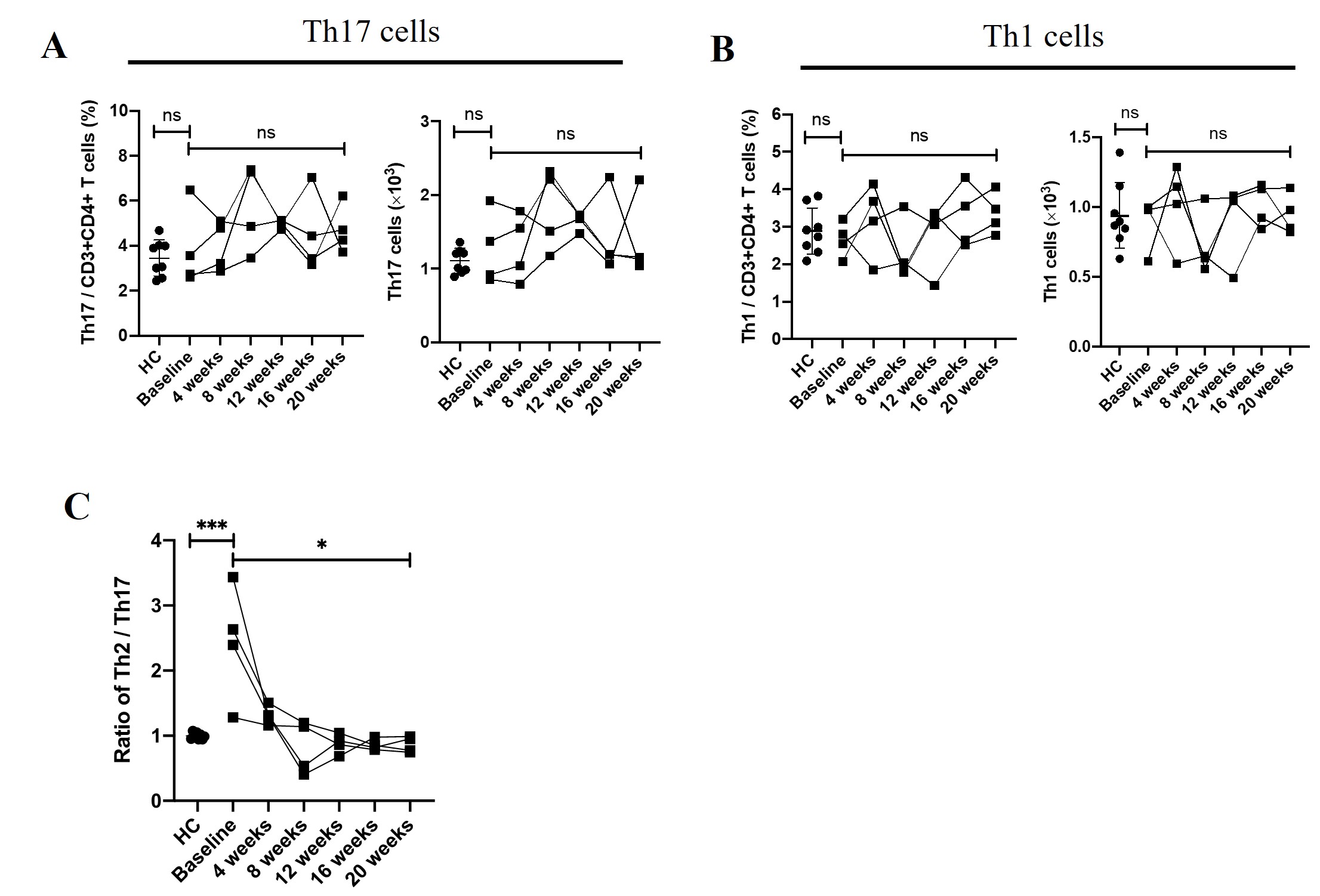

Supplement: Supplementary Figure 3 — Changes in Th1/17 cell subsets during 20 weeks of dupilumab treatment Changes in the percentage of Th17 cells (A) and Th1 cells (B) among the CD3+CD4+ T cell population and in the absolute number of Th17 cells (A) and Th1 cells (B) from baseline to 20 weeks of treatment. (C) Changes in the Th2/Th17 ratio from baseline to 20 weeks of treatment. The Wilcoxon matched pairs signed rank test or the Mann-Whitney U test was used to assess statistical significance: ****P < 0.0001; ***P < 0.001; **P < 0.01; and *P < 0.05. Each dot represents a value for each patient. [file Image_3.jpeg]
